# Effect of replacing oat fodder with fresh and chopped oak leaves on *in vitro* rumen fermentation, digestibility and metabolizable energy

**DOI:** 10.14202/vetworld.2015.1021-1026

**Published:** 2015-08-26

**Authors:** K. Rajkumar, R. Bhar, A. Kannan, R.V. Jadhav, Birbal Singh, and G. Mal

**Affiliations:** Animal Nutrition Laboratory, Indian Veterinary Research Institute, Regional Station, Palampur, Himachal Pradesh, India

**Keywords:** chopping, *in-vitro*, methane, oak leaves, oat fodder

## Abstract

**Aim:**

A study was conducted to evaluate the effect of replacing oat fodder (OF) with fresh oak leaves (FOL) or chopped oak leaves (COL) on rumen fermentation and digestibility through *in vitro* gas production technique (IVGPT).

**Materials and Methods:**

Nine different diets were prepared by mixing OF with oak leaves (either FOL or COL) in different ratios (100:0, 75:25, 50:50, 25:75, and 0:100). The rations were evaluated through Hohenheim IVGPT with 200 mg substrate and 30 ml of buffered rumen liquor. All the syringes were incubated at 39°C for 24 h in buffered rumen liquor of cattle. After 24 h, the total gas production was recorded, and the contents were analyzed for *in vitro* methane production, protozoa no. and ammonia-N.

**Results:**

Chopping (p<0.01) reduced the tannin fractions as well as non-tannin phenol. Increase in levels of oak decreased total gas production, methane, organic matter (OM) digestibility, and metabolizable energy (ME) values. The polyphenol content of the substrate did not show any significant difference on the protozoal count.

**Conclusion:**

*In vitro* studies revealed that the addition of oak leaves reduced the methane production and ammonia nitrogen levels; however, it also decreased the OM digestibility and ME values linearly as the level of the oak leaves increased in the diet. Chopping was effective only at lower inclusion levels. Further studies, especially *in vivo* studies, are needed to explore the safe inclusion levels of oak leaves in the diet of ruminants.

## Introduction

Availability of fodder among Asian countries particularly with countries like India is not adequate to meet the ever growing livestock population. There is a great need to explore new feed resources to meet this deficit without competing with food chain [1]. Tree fodders are the alternate source of small ruminant feeds to that of conventional green fodders which have the potential to mitigate the gap between demand and supply of feeds [2]. Tree fodders have similar nutritive value as that of leguminous fodders [3], which plays an important role in the nutrition of grazing animals where there is less scope of conventional fodders. Oaks (*Quercus* spp.) are one such tree fodder which is the dominant, climax tree species of the moist temperate forests of the North Western Himalayan region (NWHR). During extreme climatic condition in this agro-climatic zone, when ruminants cannot go out to graze oak leaves take cares the nutritive requirement of such animals.

Even though the oak leaves are abundantly available in the NWHR, toxicity problems exist due to sole feeding of oak leaves in the diet of ruminants [4]. Moreover, previous workers reported oak toxicity even on feeding oak leaves partially in the diets of the ruminants [5]. *Quercus* species are reported to contain the high levels of hydrolysable tannins (HT) which are the main reason for the toxicity in the livestock. HT undergo acid and microbial hydrolysis to release simple phenolics which there by cause toxicity [6,7]. Chopping of the oak leaves is the simple procedure by which the polyphenol content of the oak leaves can be reduced. Chopping helps the phenolic oxidases to get exposed with tannins which results in tannin reduction.

So, the present study was undertaken with two objectives: (i) To identify the safe inclusion level of oak leaves in replacing the conventional high quality oat fodder (OF) for feeding ruminants through Hohenheim *in vitro* gas production technique (IVGPT) (ii) To explore the additional benefit of chopping oak leaves on reducing the polyphenol content and on rumen metabolism through IVGPT.

## Materials and Methods

### Ethical approval

The study was undertaken after taking necessary approvals from the Institutional Animal Ethical Committee of the University.

### Sampling of the oak leaves

The fresh mature oak (*Quercus leucotrichophora)* leaves were manually lopped from the nearby forest area of Palampur, Kangra District, Himachal Pradesh, India. A part of the lot was chopped by a mechanical chopper to a length of 2±0.5 cm. OF were purchased from the local market. Nine different diets were prepared by mixing OF with oak leaves i.e. one diet comprising 100% OF, four diets with mixing OF with FOL (75:25, 50:50, 25:75, and 0:100) and another four diets with mixing OF with COL (75:25, 50:50, 25:75, and 0:100).

### Chemical analysis

The chemical composition of the FOL and COL, and OF were determined by the method of AOAC (2000) while fiber fractions were estimated as per the methods suggested by Van Soest *et al*. [8]. Polyphenol profile of oak leaves was estimated by the method of Makkar [9]. Total phenols (TP) and non-tannin phenols (NTP) were estimated by Folin–Ciocalteau method in combination with polyvinylpolypyrrolidone, with tannic acid as a reference standard [9]. The condensed tannins (CT) were estimated by using butanol-HCl method.

### Rumen liquor sampling

Rumen liquor was collected from two rumen cannulated cattle (body weight = 220 kg), strained through a four-layered muslin cloth and pooled together which was used as an inoculum source for *in vitro* studies. The donor animals were fed 60% wheat straw and 40% concentrate. Five different diets were prepared mixing OF with oak leaves (FOL and COL) in the ratios of 100:0, 75:25, 50:50, 25:75, and 0:100 and evaluated through Hohenheim IVGPT suggested by Menke *et al*. [10] with 200 mg substrate and 30 ml of buffered rumen liquor. All the syringes were incubated at 39°C for 24 h in buffered rumen liquor of cattle. After 24 h, the total gas production was recorded, and the contents were analyzed for *in vitro* methane production, protozoal count, and ammonia-N. *In vitro* methane production was estimated in gas-liquid chromatography (gas chromatography [GC], Nucon 5765, Nucon Engineers, New Delhi, India) equipped with a flame ionization detector. The column was of stainless steel packed with a propak-q (length 1.8 m; o.d 0.3 mm; i.d 2 mm; mesh 80-100). The analytical condition of GC was carrier gas N_2_ flow 40 ml/min, H_2_ 30 ml/min, air 300 ml/min, and temperature range at injection port was 150°C, column 60°C, and at detector was 130°C. The peak was compared with the standard (50% CH_4_ and 50% CO_2_ from SPANCAN calibration gas, Spantech, Surrey, UK) and the analysis and calculation used the Aimil chromatography data system (WINACDS, New Delhi, India).

Metabolizable energy (ME) values of samples were calculated by a formula derived by Menke and Steingass [11]. The microbial protein and digestibility were calculated with the 400 mg substrate incubated in 40 ml of buffered rumen liquor.

ME (MJ) = 2.20 + 0.136* gas (ml/200 mg DM) + 0.0057 * CP + 0.0029*EE

Microbial protein was estimated by the ­following formula:

Microbial protein (mg) = TD (mg) – (2.25 × net gas volume)

Whereas, TD = True digestible matter (substrate incubated−NDF)

The *in vitro* gas production was completed in three runs (statistical replicates) with each sample incubated in triplicate (analytical replicates).

### Statistical analysis

The analytical replicates were averaged prior to statistical analysis with each run being the statistical replicate. The data were analyzed using one-way analysis of variance procedures (SPSS base 7.5 for windows [1997]) and the difference between the treatments means were compared by Duncan’s multiple range tests. Results are presented as means and standard error of means. Treatment effects or differences were considered significant if p<0.05.

## Results and Discussion

### Chemical composition

The chemical composition of the oat fodder (OF) and oak leaves are presented in the Table-1. The chemical composition of OF at the early maturity was comparable to that reported by earlier workers [12-16]. The organic matter (OM), crude protein (CP), ether extracts (EE), nitrogen free extract (NFE), and Crude fibre (CF) content of the OF was 88.4, 14.7, 3.66, 46.47, and 23.57%, respectively. There was no significant difference between the values in the OM, CP, EE, NFE, and CF content between FOL and COL. The chemical composition of the oak leaves was comparable to that reported by earlier workers [17-21].

**Table-1:** Chemical composition of OF and oak leaves (% DMB).

Substrate component	Chemical composition					

	CP	EE	NDF	ADF	ADL	TA
OF	14.70^a^±0.13	3.66^c^±0.12	61.52^c^±0.11	35.04^c^±0.17	3.33^c^±0.07	11.60^a^±0.12
FOL	10.45^c^±0.09	4.83^a^±0.11	66.50^b^±0.12	50.16^b^±0.10	25.43^b^±0.11	4.07^b^±0.13
COL	10.87^b^±0.10	4.37^b^±0.06	69.77^a^±0.11	52.10^a^±0.18	26.10^a^±0.10	4.33^b^±0.11

*Mean of six samples each, means bearing different superscripts (a, b and c) in a column differ significantly (p<0.01), CP=Crude protein, EE=Ether extract, NDF=Neutral detergent fibre, ADF=Acid detergent fiber, ADL=Acid detergent lignin, TA=Total ash, OF=Oat fodder

### Polyphenol content

Polyphenol content (on DM basis) of the OF, and the oak leaves are presented in the Table-2. There was 10.1, 9.43, 10.3, 7.0, 11.1% reduction in the TP, NTP, TT, CT and HT values due to chopping of the oak leaves. There was a significant difference in the TP, NTP, TT, and HT content between the FOL and COL. The reduction of the polyphenol content of the oak leaves is due to the higher susceptibility of COL to oxidative enzymes and conversion of higher polymerization leading to inert phenols. The degree of susceptibility of HT to the oxidative enzyme is relatively more than the other polyphenol [22,23].

**Table-2:** Polyphenol profile of OF and oak leaves (% DMB).

Substrate component	Polyphenol profile				

	TP	NTP	TT	CT	HT
OF	1.20^c^±0.09	0.46^b^±0.03	0.74^c^±0.01	0.01^c^±0.00	0.73^c^±0.01
FOL	6.93^a^±0.08	0.53^a^±0.02	6.41^a^±0.06	1.28^a^±0.02	5.13^a^±0.04
COL	6.23^b^±0.06	0.48^b^±0.01	5.75^b^±0.06	1.19^b^±0.03	4.56^b^±0.04

*Mean of six samples each, means bearing different superscripts (a, b and c) in a column differ significantly (p<0.01), TP=Total phenol, NTP=Non-tannin phenol, TT=Total tannin, CT=Condensed Tannin, HT=Hydrolysable tannin, OF=Oat fodder, COL=Chopped oak leaves, FOL=Fresh oak leaves

### Total gas production

**Graph-1:**
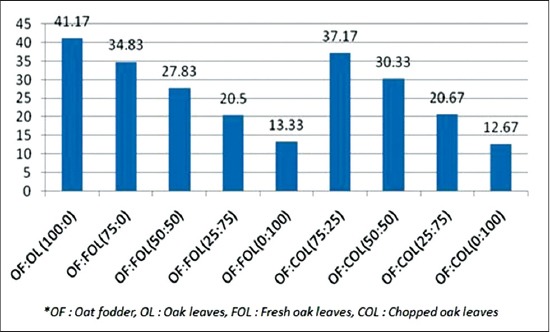
Total gas (ml/200 mg dry matter).

The total gas production per 200 mg substrate was higher at the 100% oat concentration (41.17 ml). Increase in levels of oak decreased total gas production (ml/200 mg DM) (Graph-1) at a decreasing rate. Gas production was more in the COL group than the FOL at the same ratio. There was a significant difference (p<0.01) between the values of 25% oak leaves in COL and FOL. The gas produced in the syringes is largely due to acetate and butyrate, and lower gas production is associated with propionate production. Easily fermentable carbohydrates yield higher propionate therefore leading to less gas. The gas production is negatively related with the neutral detergent fiber (NDF) content and positively with the starch content. Tannins especially HT at higher levels are toxic to the rumen microbes, therefore, leading to less gas production [24].

### Methane

**Graph-2:**
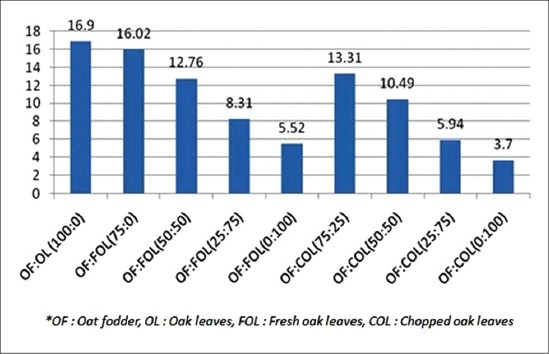
Methane production (ml/200 mg dry matter).

The methane production was maximum in the 100% OF level (16.9 ml/200 mg). As the concentration of oak leaves increased in the substrate, the methane production decreased (Graph-2). The effect on methane production was parallel to decreased total gas production. Even at the 25% inclusion level of oak, there were 5.2% and 21.24% reduction of methane in fresh and chopped oak, respectively. There was significant (p<0.01) difference between the methane produced in COL and FOL group (except at 25% oak). There was less production of methane in COL than the FOL of the same ratio. Studies using CT-containing forages such as big trefoil (*Lotus peduncalatus*, 53 g/kg CT), sulla (*Hedysarum coronarium*, 27-68 g/kg CT), red clover (*Trifolium pretense*, 3 g/kg CT), and *Sericea lespedeza* (*Lespedeza cuneata*, 177 g/kg CT) reported reductions in CH_4_ emissions [25-28]. Tannins present in different plants such as *Calliandra calothyrsus* [29] and *Onobrychis viciifolia* [30] and *Populus deltoides* [31] reduced methane production under in *vitro* conditions. Similar results were reported by Woodward *et al.*, [26] that CT containing *H. coronarium* forage reduced methane production per kg DM intake (19.5 vs. 24.6 g) in grazing cows. Similarly, sheep fed *L. corniculatus* silage reduced methane production [32]. Waghorn *et al*. [25] reported 16% reduction in methane production in lambs fed on CT containing *Lotus pedunculatus* (lotus).

### Microbial protein synthesis

Microbial protein synthesis was estimated through equations with the help of total gas production. Microbial protein was higher at the 100% OF (164.69 mg), however, as the oak leaves percentage increased in the substrate incubated the production of microbial protein decreased linearly. There was no significant difference between the values in COL and FOL. The linear reduction in the microbial protein was due to the toxic effect of the polyphenols to the rumen microbes [33]. For microbial protein synthesis synchronization of the rate of degradation of N and carbohydrate components in the rumen is important for efficient utilization of rumen ammonia nitrogen. Therefore, there is a reduction of microbial protein synthesis with respect to ammonia nitrogen.

### True dry matter (DM) and OM digestibility

**Graph-3:**
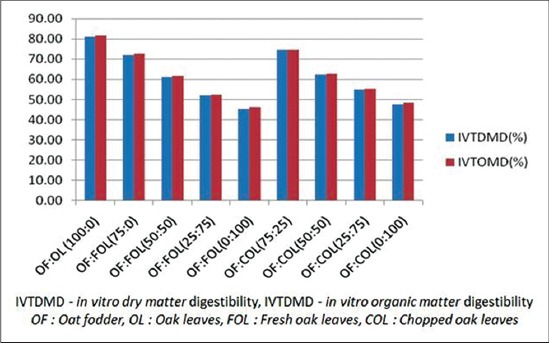
Effect of replacing oat fodder with fresh or chopped oak leaves on *in vitro* digestibility.

Both the DM and the OM digestibility were higher in the 100% OF (81.14 and 81.73%, respectively). Both DM and OM digestibility followed the same trend, i.e. when the percentage oak leaves increased the digestibility decreased (Graph-3). There was a significant difference between the values at 50% oak leaves in COL and FOL. The reduction in the digestibility is attributed to the high tannin and lignin content in the diet [34,35]. Tannins reduce digestibility by reducing the activity of rumen microbes, by binding with rumen enzymes, or by binding with feed components [36]. Particularly in case of tree leaves, tannins are present in NDF and acid detergent fiber (ADF) fractions in certain amounts which are tightly bound to the cell wall and cell proteins and it is believed to be involved in decreasing digestibility [37].

### Ammonia nitrogen

The ammonia nitrogen (mg/30 ml) in the different dietary combination is presented in Table-3. The ammonia nitrogen (mg/30 ml) produced was maximum in the 100% OF (6.6). There was a significant difference (p<0.01) in the ammonia nitrogen production in all the ratios between COL and FOL groups (except at 25%). Tannins are known to bind with proteins, which is the key reason for the reduction of rumen ammonia concentration [38]. Many authors have indicated that the principal effects of tannins in ruminal fermentation include a reduction in proteolysis of dietary protein and subsequently lower concentrations of ammonia in rumen fluid [39,40]. All ammonia concentrations were higher than the 100 mg/L reported by Van Soest *et al*. [8] as optimal for the efficiency of amino acid synthesis and microbial growth. Although that value might depend on a number of factors, such as the amount of available fermentable energy [41], ammonia concentrations were probably adequate for optimal rumen fermentation in all cases.

**Table-3:** Effect of OF and oak leaves at various combinations on *in vitro* gas production and rumen fermentation.

Combination	Microbial protein (mg)	Ammonia nitrogen (mg/30 ml)	Protozoa (×10^4^)/ml	ME (MJ/kg DM)	Methane ml/200 mg
Oat (100%)	164.69^a^	6.60^a^	1.74^ab^	7.89^a^	16.90^a^
OF+FOL					
Oat (75%)+oak (25%)	155.22^ab^	6.20^b^	1.85^ab^	7.03^c^	16.02^a^
Oat (50%)+oak (50%)	143.37^bc^	5.13^e^	2.37^a^	6.07^e^	12.76^b^
Oat (25%)+oak (75%)	133.06^c^	5.23^de^	2.11^a^	5.07^f^	8.31^d^
Oat (0%)+oak (100%)	133.14^c^	5.23^de^	1.56^ab^	4.09^g^	5.52^e^
OF+COL					
Oat (75%)+oak (25%)	156.19^ab^	5.60^c^	1.59^ab^	7.34^b^	13.31^b^
Oat (50%)+oak (50%)	138.83^c^	5.47^cd^	1.89^ab^	6.41^d^	10.49^c^
Oat (25%)+oak (75%)	144.30^bc^	5.10^e^	2.18^a^	5.09^f^	5.94^e^
Oat (0%)+oak (100%)	144.40^bc^	4.70^f^	1.22^a^	4.00^g^	3.70^e^
SEM	2.36	0.11	0.10	0.26	0.90
p significance	p<0.01	p<0.01	NS	p<0.01	p<0.01

*Mean of six samples each, Means bearing different superscripts (a, b, c, d, e, f and g) in a column differ significantly (p<0.01), OF=Oat fodder, SEM=Standard error of means, DM=Dry matter, COL=Chopped oak leaves, FOL=Fresh oak leaves, ME=Metabolizable energy

### Protozoal count

The number of protozoa in the different ratio of oat: Oak is represented in the Table-3. The polyphenol content of the substrate did not show any significant difference on the protozoal count. Similar results were reported with *Q. leucotrichophora* [20]. Tavendale *et al*. [42] suggested that inhibition of growth of methanogens is due to the bacteriostatic and bactericidal effects of CT. Since some of the methanogens are ecto- and endo-symbiotically associated with protozoa, a reduction in methanogens would probably affect the protozoal population [43]. The effects of tannin on the protozoal number are conflicting, some authors claim in the reduction of protozoal number with tannin supplementation, but others claim no effect. Monforte-Briceno *et al*. [44] studied the defaunating properties of 15 tree fodders containing tannins, but the inhibitory effect on protozoa was observed in *Acacia farnesiana*, *C. calothyrsus* and *Lysiloma latisiliquum*. Tannins present in tanniferous plants are not equally efficient in reducing the protozoal count.

### ME values

The calculated ME (MJ/kg DM) values were higher in the 100% OF group (7.9). A similar trend was seen as that of the gas production in oak groups, i.e. as the % oak increased in the substrate, the ME values decreased. The ME values of the fresh oak group was 7, 6.1, 5.07 and 4.09 MJ/kg DM and in chopped oak group was 7.34, 6.41, 5.09 and 4 MJ/kg DM at 25, 50, 75 and 100% respectively. The ME values of 100% FOL and COL were also estimated by Ajith [20], ME value of FOL and COL reported by him were 5.76 and 5.52 MJ/kg DM. It is well-known fact that NDF, ADF and CT are negatively associated with ME of feedstuffs [45].

## Conclusion

The current *in vitro* study revealed that the addition of oak leaves reduced the methane production and ammonia nitrogen levels; however, it also decreased the OM digestibility, microbial protein synthesis and ME values linearly. Oak tannins didn’t have any effect on the protozoal number. Even though, there was a reduction in polyphenol content due to chopping, it was effective only at lower inclusion levels (i.e. 25% and 50%). However, comprehensive *in vivo* studies with animal hosts need to be undertaken to evaluate the sustainability of oak leaves supplementation to mitigate rumen methanogenesis without detrimental effects on the animal as a whole.

## Authors’ Contributions

RB and AK planned and supervised the entire research work. KR and RJ carried out the experimental work and laboratory analysis. BS and GM prepared the manuscript along with data analysis. All authors read and approved the final manuscript.
